# Rock Slope Stability Analysis Using Terrestrial Photogrammetry and Virtual Reality on Ignimbritic Deposits

**DOI:** 10.3390/jimaging10050106

**Published:** 2024-04-28

**Authors:** Tania Peralta, Melanie Menoscal, Gianella Bravo, Victoria Rosado, Valeria Vaca, Diego Capa, Maurizio Mulas, Luis Jordá-Bordehore

**Affiliations:** 1 Faculty of Engineering in Earth Sciences (FICT), ESPOL Polytechnic University, Gustavo Galindo Campus, Guayaquil 090101, Ecuador; tgperalt@espol.edu.ec (T.P.); mmenosca@espol.edu.ec (M.M.); gmbravo@espol.edu.ec (G.B.); vrosado@espol.edu.ec (V.R.); vpvaca@espol.edu.ec (V.V.); dcapa@espol.edu.ec (D.C.); 2 Department of Engineering and Fiel Morphology, Polytechnic University of Madrid, 28040 Madrid, Spain

**Keywords:** rock mass, 3D virtual outcrop models, point cloud, kinematic analysis, photogrammetry, metaverse

## Abstract

Puerto de Cajas serves as a vital high-altitude passage in Ecuador, connecting the coastal region to the city of Cuenca. The stability of this rocky massif is carefully managed through the assessment of blocks and discontinuities, ensuring safe travel. This study presents a novel approach, employing rapid and cost-effective methods to evaluate an unexplored area within the protected expanse of Cajas. Using terrestrial photogrammetry and strategically positioned geomechanical stations along the slopes, we generated a detailed point cloud capturing elusive terrain features. We have used terrestrial photogrammetry for digitalization of the slope. Validation of the collected data was achieved by comparing directional data from Cloud Compare software with manual readings using a digital compass integrated in a phone at control points. The analysis encompasses three slopes, employing the SMR, Q-slope, and kinematic methodologies. Results from the SMR system closely align with kinematic analysis, indicating satisfactory slope quality. Nonetheless, continued vigilance in stability control remains imperative for ensuring road safety and preserving the site’s integrity. Moreover, this research lays the groundwork for the creation of a publicly accessible 3D repository, enhancing visualization capabilities through Google Virtual Reality. This initiative not only aids in replicating the findings but also facilitates access to an augmented reality environment, thereby fostering collaborative research endeavors.

## 1. Introduction

Slope stability has long been a focal point of study, particularly concerning the safety of those utilizing structures such as roads that traverse these slopes. 

The assessment of stability of rocky slopes demands meticulous attention to field data collection, as the interpretation of geomechanical data pivotal to understanding slope instability relies on it [1,2]. Slopes adjacent to highways may exhibit various failure types contingent upon mass rupture or discontinuities [3]. 

Discontinuities near the surface can precipitate kinematic failures, such as wedging, planar slips, or toppling, underscoring the necessity of stability evaluations to ensure user safety [4,5].

Despite technological advancements, direct field data collection on rocky slopes remains indispensable for kinematic analysis. Geomechanical observation consolidates essential observations crucial for slope characterization [6,7]. 

Multiple methodologies, grounded in the physical and mechanical attributes of rocks, are employed to refine slope characterization [8]. Terrestrial photogrammetry was performed using a digital camera, and predominantly was used to discern geometric attributes of challenging high-altitude slopes. It was facilitated by software-processed digital images obtained from various vantage points, presenting a cost-effective alternative [9,10,11,12].

Through this technique, the intention is to combine field observation and, later, kinematic analysis to establish how stable the studied rock masses are [13].

The overarching aim of this study is to assess rock mass stability via mapping and processing techniques, elucidating the advantages of each applied methodology.

**Figure 1:**
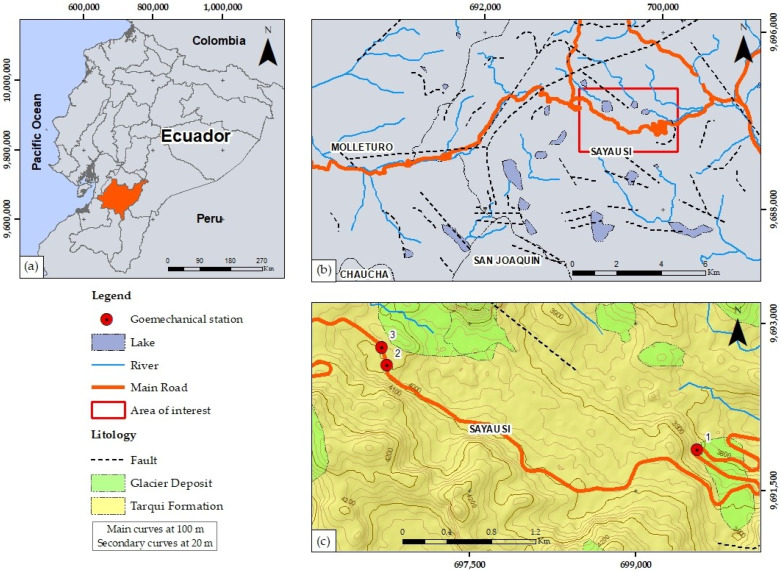
General aspects of the study area: (**a**) location of the study are, (**b**) analyzed area with the main structural lineament using the methodology of Villalta et al., 2022 [10], (**c**) geological map of the study area in the Tarqui Formation (PT) and Glacier Deposit [16].

The study site lies along the Cuenca–Molleturo Road, within Cajas National Park, Azuay province, southern Ecuador, spanning abscissas 28 + 060 − 33 + 200 (Figure 1).

This region, nestled in the Ecuadorian Andean moorland within the western Andes mountain range, experiences a cold and humid climate, with an average annual rainfall of 1106 mm and an average temperature of 5.78 °C [14].

The deposits within this area are attributed to the Tarqui Formation, characterized by poorly consolidated and deeply altered primary volcaniclastic fall deposits, with a maximum thickness of 300 m [15].

This study employs a relatively novel approach to remotely ascertain slope characteristics, mitigating the inherent dangers associated with direct field acquisition. Geomechanical stations have been strategically positioned to define the characteristics of the massif, followed by validation of processed data conducted in the office.

## 2. Materials and Methods

### 2.1. Information Collected in the Field

Analyzing the stability of rock slopes within jointed rock masses at shallow depths requires a systematic approach to evaluate the orientation and strength of discontinuities such as joints, strata, and faults, which directly influence their stability [17,18]. The portions of the slope in direct contact with the surface are most susceptible to geological and climatic processes, gradually altering their topography [19].

**Figure 2:**
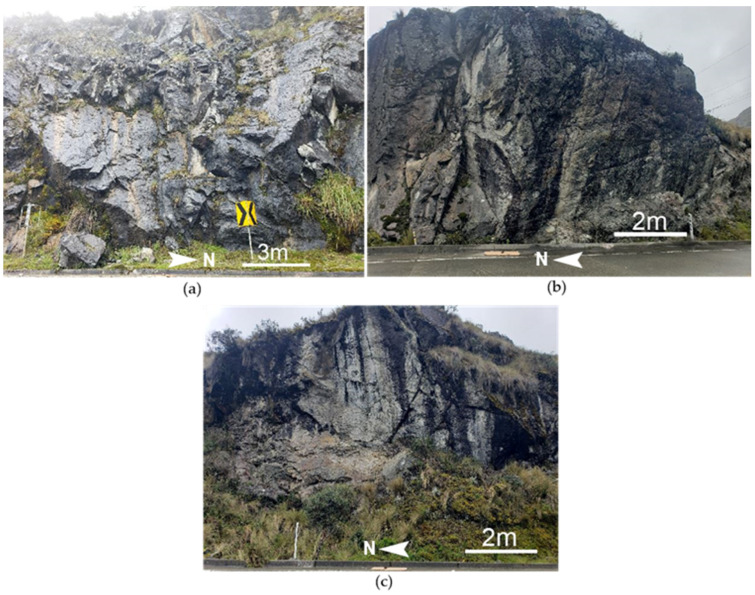
General view of slopes analyzed: (**a**) Slope 1, (**b**) Slope 2, (**c**) Slope 3.

Traditionally, field data collection involves deploying geomechanical stations at each slope or site (see Figure 2). However, contemporary terrestrial photogrammetry provides a safer means for engineers to characterize slopes, supplementing direct observations made in the field.

The Cajas massif exhibits a notably irregular topography, ranging from 3160 m above sea level (in the Llaviucu zone) to 4500 m above sea level (at Cerro Arquitectos), featuring expansive plains and valleys [20].

Field campaigns were conducted in April 2023, during the rainy season of the Ecuadorian Sierra. Detailed information essential for characterizing each slope—such as roughness, persistence, slope geometry, and orientation—was diligently recorded in a field notebook. Additionally, orientation measurements of discontinuities were obtained (Table 1).

The resistance to simple compression was assessed using a Schmidt hammer [21,22].

The array of technological tools available for acquiring and processing field data is diverse, encompassing photogrammetry, structure from motion, point cloud evaluation, and 3D reconstruction, all instrumental in extracting crucial information such as geometry, location, surface characteristics, and kinematic analysis [23,24,25]. To generate the 3D point cloud, photographs captured from cameras were utilized across each of the rocky slopes, complementing data acquired through direct observation. This rapid and cost-effective methodology does not necessitate professional-grade images [26]. The images for the photogrammetric analysis were taken with an iPhone 13 mobile phone without the GPS location. To orientated the images, we used a local coordinate system with 3 points in a table. The images were horizontally orientated with the Y axis towards north.

This approach not only enhances safety, as physical contact with the slope can pose risks, but also mitigates the costs and time associated with conventional data collection methods. Furthermore, it reduces the potential bias introduced by inaccessible areas of the slope during data collection [27,28].

### 2.2. 3D Point Cloud Generation and Virtual Reality Approach

Remote sensing for modelling rock masses from 3D point clouds has experienced significant growth due to technological advancements [26], thereby influencing indirect data collection strategies for rocky slopes that are challenging to access. The quantity of photographs captured for each slope varies, with a considerable number required to yield optimal results [29,30].

Once processed, the images were exported as files and loaded into Cloud Compare Software version 2.12.4, a tool designed for handling and comparing 3D point clouds, Refs. [31,32], and facilitating the extraction of parameters from slopes that are hard to reach in the field.

This process was repeated for each slope, generating multiple high-quality point clouds to closely approximate reality.

For gathering dip angle and dip direction data, the FieldMove Clino app by Petroleum Experts—a compass-clinometer for smartphone data capture—was employed. This application enables orientation measurements of geological structures like faults, joints, begging, lineation, slickensides, fold axis, and geological mapping [33,34,35,36,37].

**Figure 3:**
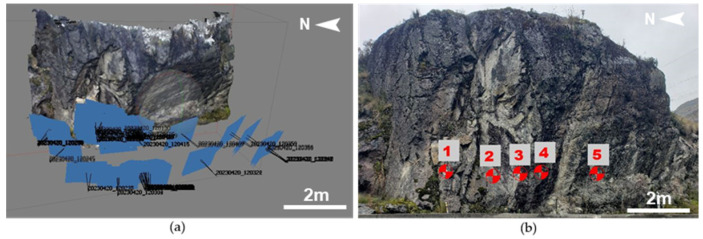
Slope 2: (**a**) point cloud obtained with Agisoft, (**b**) control points for orientation.

Measurements using the Clino application were taken at various points on the slopes, with specific locations identified on certain planes for subsequent comparison, ensuring that any differences between the two measurements did not exceed 5°, as recommended [26,37,38]. Following image processing, the outcome was a 3D model offering enhanced visualization of each slope’s characteristics, particularly those challenging to access (see Figure 3).

### 2.3. Discontinuities

The orientation of joints in rock masses, commonly referred to as discontinuities, is important for assessing the stability and risk of the slope [28,39]. 

The discontinuities generate sets that are observed in a rock slope and generally tend to have a similar morphology and orientation [40]. The data obtained manually in the field were used and compared with the data obtained using Cloud Compare 2020 software with the virtual compass tool.

The orientations resulting from the measurements are shown in Table 2, where the differences between field data and terrestrial photogrammetry data can be observed, along with the number of sets present in each rocky slope.

Obtaining representative information about the discontinuities is crucial for defining potential kinematic mechanisms that the slope could exhibit [7,41].

The features of the discontinuities were manually obtained in safe and accessible areas of the studied slopes. Measurements in areas difficult to access were carried out once the 3D point clouds were obtained for greater security.

From the orientations belonging to each family of discontinuities observed in the field, stereograms were produced, graphically representing these families and enabling determination of their most probable failure mode.

### 2.4. Empirical Analysis

The structures present in a rock mass exert the greatest control over its behavior; therefore, classification systems relate low strength directly to highly fractured rock masses [42]. The classification systems for the slopes of the present study are SMR and Q-Slope, which are the most used in engineering practice [43].

RMR classification comprises field data collection and post-desk processing; it is mainly based on spacing information and joint conditions [44]. As for the Q-Slope classification, it varies on a logarithmic scale from 0.001 to a maximum of 1.000 [45].

**Slope Mass Rating (SMR)** [46] derives from the Rock Mass Rating [47]; the Slope Mass Rating is obtained by adjusting factors that depend on the joint–slope relationship and the excavation method applied [48,49,50]. In Equation (1), the correction factors for the SMR are shown, considering aspects of the type of excavation and the orientations.

(1) $$SMR=RMR+\left(F1\times F2\times F3\right)+F4$$

**Q-slope classification.** The Q-slope is a recently developed rock mass classification that is specific to rock slopes [51]. It uses six parameters: RQD, Jn, Jr, and Ja, which are the same as in the Q index, but it introduces a correcting factor named the O-factor depending on the apparent stability of the slope. The Jw and SRF of the Q system are now called the Jwice, which includes other aspects, and the SRF is obtained from three different methods, selecting the most unfavorable [51].

(2) $$Q_{slope}=\frac{RQD}{J_{n}}\times {\left(\frac{J_{r}}{J_{a}}\right)}_{O}\times \frac{Jwice}{SRFslope}$$

According to [26], it is not superfluous to carry out the calculation to determine the angle of maximum slope in which the slope can be considered a stable slope.

$$\beta =20\log_{10}Q_{slope}+65{}^{\circ}$$

### 2.5. Kinematic Analysis 

Generally, kinematic analysis is carried out on slopes in which the discontinuities induce the movement of blocks, and the failure may be planar, wedge, or toppling depending on the conditions that occur in the rock mass.

The different geological structures present in a rock mass affect its quality and stability [52]. However, it is the discontinuities that govern its stability, so from the kinematic analysis it can be examined [53].

Kinematic analysis is very useful to determine the sites that are prone to failure according to their directions and the joints they have [54], as well as to determine the failure mechanism through a relationship between structural geology and stability conditions [55]. The angular relationships between the discontinuities and the dip of the rock formations are applied to determine the potential for failure and its type [56].

In the present study, the kinematic analysis was carried out using the data collected in the field through the DIPS software [57]. The orientations of the discontinuities can be represented in a stereogram in the form of great circles, poles, or vectors of immersion. Similarly, the families of discontinuities were identified in the field along with their dip/dip direction characteristics.

To apply the strength criteria of discontinuities [58], it was necessary to assume some parameters, with the basic friction angle φb = 30°, specific weight of the rock 0.028 MPa [59], and the coefficient of roughness JRC = 8 assumed for all slopes. Rock strength was obtained by approximation with a Schmidt hammer as mentioned above. In this case, we used manual measurements and also manual picking in the point cloud models instead of synthetic or automatic ones [60].

The angle φr was obtained from the Barton–Choubey relation [61], taking as a range 26 to 31 degrees [26].

## 3. Results

### 3.1. Discontinuity Set Analysis

**Figure 4:**
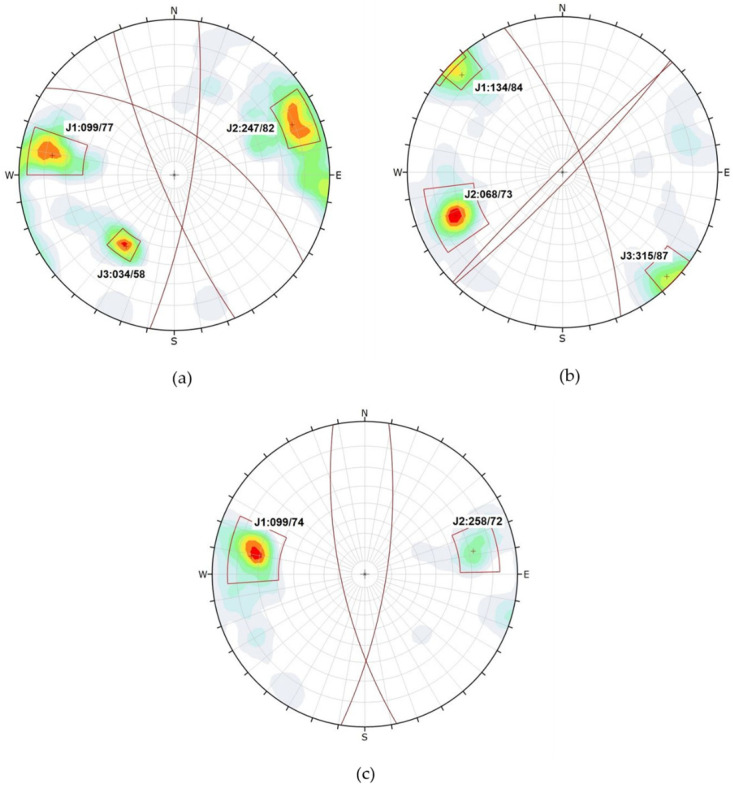
Discontinuities set in each slope: (**a**) Slope 1, (**b**) Slope 2, (**c**) Slope 3.

Once the point cloud was processed, two to three families were identified (Figure 4) and used for analysis. It was deemed necessary to conduct a manual analysis of the discontinuities. In the 3D model generated with the Agisoft metashape 2.1.1 software (in addition to the data obtained in the field with the digital compass), between 148 and 200 planes were measured in each of the slopes. In slope number 3, only two families of discontinuities were identified.

Table 2 presents the main joint sets of each slope, along with the number of measurements taken from the 3D point cloud manually using the Cloud Compare software.

The measurements taken with the Clino mobile application and those obtained with the Cloud Compare Software vary by a maximum of 8°, considering that multiple measurements of the same planes were taken in the field to minimize execution errors.

Similarly, as mentioned in Section 2.2, the control points located on each slope help reduce the discrepancy between field measurements and those conducted in the office. It has also been analyzed that the distribution of measurements is influenced by climatic zones [56].

### 3.2. Rock Mass Classification Systems

**Slope Mass Rating (SMR).** The basic RMR value obtained for each slope indicates that its quality ranges from poor to medium, with values ranging from 33 to 53.

Regarding the SMR, after applying the correction factors, qualities ranging from very poor to normal were obtained for the three slopes (Table 3). Considering the orientations of the discontinuities, the potential failure mode for each slope was determined.

**Q-slope.** In the same manner as conducted for the SMR system, the Q-slope index for each slope under study was also analyzed. Initially, there is competent rock, but it may nonetheless pose stability risks.

The environment where the slopes are situated is humid and experiences low temperatures, leading to varying degrees of alteration. Roughness ranges from flat to irregular at the macroscopic level and rough and irregular overall. Given that the slopes are situated alongside a road, they align well with the Q-slope classification methodology.

The results obtained through this system indicate that Slope 2 is on the borderline between stable and uncertain, while Slopes 1 and 3 can be deemed stable (Table 4).

The results obtained with the Q-slope System differ somewhat from those determined with the SMR System, as the slopes appear to be mostly stable according to the Q-slope method. The Q-slope System overlooks certain features, such as the excavation mode of rock masses, which could account for the discrepancy in results, as noted by Delgado et al. (2023) [26].

### 3.3. Kinematic Analysis

Table 5 presents the results of the analyses for the different structural measurements, presented as percentages for each slope and its discontinuity set. This percentage represents the proportion of structural measurements posing a high risk of kinematic failure (planar, wedge, or flexural toppling). The orientations of each of the slopes are (in DipDir /Dio mode): Slope 1 (115/78), Slope 2 (262/68), and Slope 3 (245/77).

In Figure 4, stereograms of the discontinuities with their respective families are depicted. A higher concentration of points is observed in families J1 and J2 for Slope 1, whereas for Slope 2, family J2 is most prominent, and for Slope 3, family J1 predominates.

**Figure 5:**
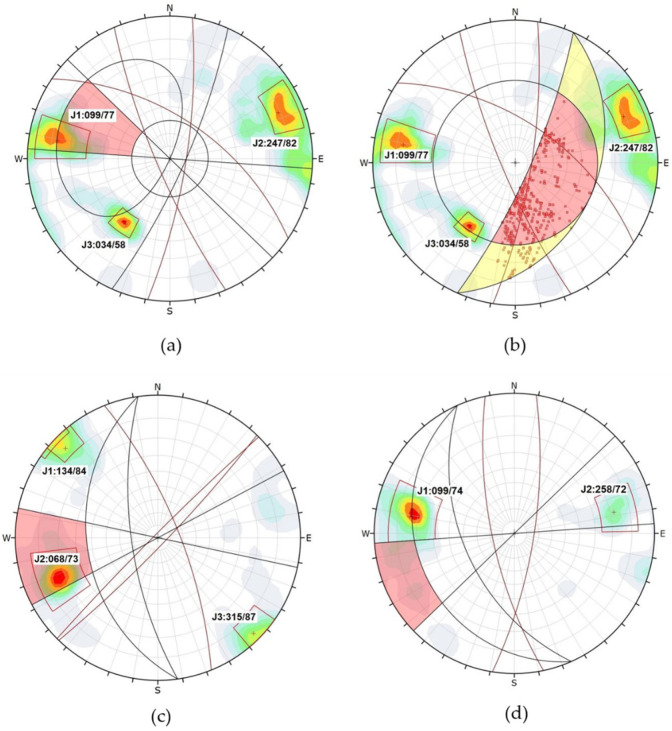
Kinematic analysis: (**a**) Slope 1 with possible planar failure J1, (**b**) Slope 1 with possible failure J1–J2, (**c**) Slope 2 with possible flexural toppling failure J2, (**d**) Slope 3 with possible flexural toppling failure J1 (although the percentage is low, it has been considered).

Meanwhile, Figure 5 illustrates the results for each potential failure mechanism in the different slopes. For Slope 1, the most probable failure modes are planar and wedge due to the arrangement of the planes. In the case of Slopes 2 and 3, failure due to flexural toppling is possible.

### 3.4. Slope Visualization in the Metaverse

**Figure 6:**
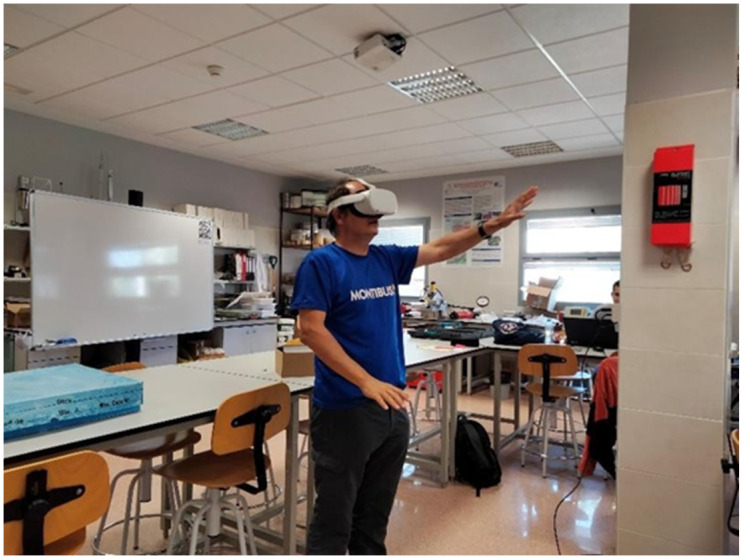
Results can be analyzed on the web and in a semi-immersive way using VR googles.

In research reports and papers, it is often possible to replicate the experiment using the geometry and geotechnical parameters presented in the methodology and results sections of the work. In this study, we take a step further by uploading the results to a repository of 3D images on the web. This repository can be viewed in semi-immersive mode through virtual reality with the help of VR goggles (Figure 6). Below, you will find the links to the three slopes in the accessible 3D repository (Table 6). We have used a commercial online repository that allows the option to view the images in VR mode in obj format. The methodology used by the platform is not accessible to the user.

## 4. Discussion

To integrate various methodologies, survey campaigns were conducted using geomechanical stations, enabling the analysis of rocky massif slope stability in specific sections of the road under study.

Fieldwork was complemented with photograph processing in software, resulting in a cloud of base points for measurements and characteristics of inaccessible slope sections. When measuring orientations (DipDir/Dip) using control points in the field with the FieldMove Clino application and in the Cloud Compare Software, errors were less than 10°, which was deemed acceptable for this study.

Based on the analysis of Slope 1, a potential planar failure caused by the J1 set was identified, with the same set potentially causing a wedge failure at its intersection with the J2 discontinuity set. The SMR methodology indicates a possible planar failure in J1 and J2, with partial stability between these two families. However, the Q-slope methodology suggests stability, possibly due to aspects not considered in the SMR methodology.

For Slope 2, which has the smallest dip angle among the studied slopes, planar failure in its J2 joint family and flexural toppling in its J1 family were identified. However, wedge failure was not observed. Analysis with the Q-slope methodology was inconclusive due to being in a transition zone, emphasizing the importance of comparing analyses using different methodologies.

Slope 3 exhibited two possible failure types: planar failure in the J1 and J2 joints and flexural toppling, primarily in J1. While the J1 family could experience flexural toppling, its percentage of critical planes (12.5%) was relatively lower compared to other slopes.

The SMR method suggested partial stability under certain failure conditions, but corroboration with other methodologies revealed unstable or stable conditions more conclusively. The combination of different analysis methods is crucial for determining stability and potential failure modes.

Results from SRM and kinematic analysis were relatively similar, whereas the Q-slope method yielded different and sometimes inconclusive results. This disparity could be attributed to the Q-slope methodology’s focus on new slopes, whereas the studied slopes are not new or recently excavated.

Table 7 summarizes the stability results for each slope, along with visual observations from field assessments.

## 5. Conclusions

In this study, various methodologies for data collection and stability analysis in rock masses have been integrated. Undoubtedly, a smartphone application can never fully replace the manual measurements made with a traditional compass. However, to keep up with technological advancements, conducting measurements of slope direction, dip, and discontinuities using such applications represents a more resource-efficient approach.

Moreover, combining terrestrial photogrammetry using a digital camera for remote measurements in inaccessible areas not only reduces costs but also enhances safety for field engineers. Additionally, it contributes to the preservation of areas like Cajas, a protected region where disturbance of fauna and landscape is prohibited.

Although all three stability analysis methodologies were applied to the three slopes, the Q-slope method, due to its omission of factors such as discontinuity orientation, yielded somewhat different and even inconclusive results (as seen in the case of Slope 2). Therefore, it is recommended to always conduct analyses by combining various methodologies to achieve a clearer and more realistic understanding.

Considering the results, it could be concluded that all three slopes are susceptible to different types of failure. Each slope exhibits susceptibility to planar failure, with Slope 2 additionally prone to flexural toppling within its J1 set, and Slope 3 also susceptible to wedge failure.

Furthermore, while combining different methodologies for rock mass stability analysis without conducting a limit equilibrium analysis may seem inconclusive, it provides valuable insights into preliminary failure mechanisms of slopes.

## Figures and Tables

**Table 1:** General information of slopes.

Slope	Coordinates (WGS 84)		Height (m)	DIP Slope (°)	Type of Slope
	East	North			
1	699,542	9,691,876	12	78	Excavated
2	696,756	9,692,613	6.8	68	Excavated
3	696,710	9,692,774	7.5	77	Excavated

**Table 2:** Orientation of the discontinuities obtained by combining manual measurements made with the Clino application in the field and the virtual compass of Cloud Compare.

Slope N°	Joint Sets Identified (Orientation DipDir/Dip)			Number of Measurements
	J1	J2	J3	
1	099/77	247/82	034/58	148
2	134/84	068/73	315/87	165
3	099/74	258/72	N.I.	200

**Table 3:** SMR obtained and correction factors for each slope.

Slope N°	RQD	UCS (MPa)	RMR	Type of Failure (Joint Sets)	Joint Sets Identified (Orientation DipDir/Dip)				SMR	Stability
					J1	J2	J3	J4		
1	90–100	50	53	Planar (J1) Wedge (J1–J3) Toppling (J1) Toppling (J2) Planar (J2)	0.7 0.15 0.15 0.15 0.15	1 1 1 1 1	−50 −60 −25 −25 −6	8 8 8 8 8	26 51.8 57 57 39	Unstable Partially Stable Partially Stable Partially Stable Unstable
2	70–80	40	33	Wedge (J1–J3) Toppling (J1) Planar (J1) Planar (J2)	0.15 0.15 0.15 0.7	1 1 1 1	−25 −25 −50 −50	8 8 8 8	40.5 37 38 10	Partially Stable Unstable Unstable Completely Unstable
3	90–100	50	37	Wedge (J1–J2)	0.15	1	−25	8	41	Partially Stable

**Table 4:** Base parameters and Q-slope calculations for each slope.

Slope N°	RQD	Jn	Jr	Ja	O-Factor	Jwice	SRF	Q-Slope	β (°)	Stability
1	91	12	4	1	0.75	0.5	15	0.76	63	Stable
2	77	12	4	4	0.75	0.5	15	0.16	49	Transition
3	91	6	7	7	1	0.6	5	0.39	57	Stable

**Table 5:** Kinematic analysis results and their percentage of critical planes.

Slope N°	Type of Failure	Discontinuity Set	Stability	% of Critical Planes
1	Planar Flexural toppling Wedge sliding	J1 - J1–J2	Unstable Stable Unstable	44 - 36.5
2	Planar Flexural toppling Wedge sliding	- J2 -	Stable Unstable Stable	- 96.43 -
3	Planar Flexural toppling Wedge sliding	- J1 -	Stable Unstable Stable	- 12.5 -

**Table 6:** Slope scan and visualization.

Slope N°	www Link	QR Code
1	https://sketchfab.com/3d-models/ta-lud-1-1d94292e7d8c4828832190167a8d984f (accessed on 25 April 2024)	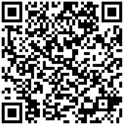
2	https://sketchfab.com/3d-models/ta-lud-2-4683ef56d7eb44b99034b57a02951de3 (accessed on 25 April 2024)	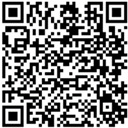
3	https://sketchfab.com/3d-models/ta-lud-3-692c9f0a5f594aa8aafdf2d9baefa7b5 (accessed on 25 April 2024)	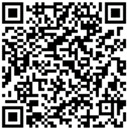

**Table 7:** Summary of stability results for the studied slope.

Slope N°	SMR	Q-Slope	Kinematic	Visual
1	Unstable	Stable	Unstable	Unstable
2	Unstable	Transition	Unstable	Unstable
3	Unstable	Stable	Unstable	Unstable

## Data Availability

The data presented in this study are available on request from the corresponding author.
